# Influence of Stress Connected with Moving to a New Farm on Potentially MAP-Infected Mouflons

**DOI:** 10.1155/2014/450130

**Published:** 2014-03-04

**Authors:** Radka Pribylova-Dziedzinska, Iva Slana, Jiri Lamka, Ivo Pavlik

**Affiliations:** ^1^Veterinary Research Institute, Hudcova 70, 621 00 Brno, Czech Republic; ^2^Department of Pharmacology and Toxicology, Faculty of Pharmacy in Hradec Kralove, Charles University in Prague, Heyrovskeho 1203, 500 05 Hradec Kralove, Czech Republic

## Abstract

There is no European legislation concerning paratuberculosis that requires that imported animals be kept in quarantine and commonly they are directly released into areas with other animals. In this study, detection of latent infection of paratuberculosis in healthy mouflons previously diagnosed as paratuberculosis-free, but originating from a real time quantitative PCR- (qPCR-) positive herd, occurred after their transport to a new farm. During a twelve-day quarantine period, all mouflons irregularly shed *Mycobacterium avium* subsp. *paratuberculosis* (MAP) in faeces, and in a small number of cases also in milk. After the animals were released from quarantine, MAP was detected for a further two days, after which, testing was negative, except in one case. Therefore, the stress connected with transport, novel environment, dietary change, or limited area with high density of animals might have contributed to the induction of paratuberculosis and the shedding of MAP from the animals, previously diagnosed as MAP-negative. According to these results, the keeping of imported animals in quarantine and their examination for MAP presence not only before the transport but also afterwards should be recommended. The designation of a particular area of a farm as a quarantine enclosure could help to mitigate the impact of stress caused by a confined space with a high density of animals.

## 1. Introduction

Paratuberculosis or Johne's disease (JD), caused by *Mycobacterium avium *subsp. *paratuberculosis* (MAP), is a disease affecting mainly domestic but also wild ruminants, including mouflons (*Ovis musimon*; wild sheep) [1–3]. In wild ruminants, paratuberculosis often manifests subclinically without overt symptoms which makes the disease more concerning from an epidemiological point of view [3, 4]. During the subclinical as well as clinical phase of the infection, the animals spread the agent into the environment by faecal shedding, where it can act as a source of infection for other animals. Young animals are the most susceptible to infection [5].

According to European veterinary legislation, animals intended for transport across different regions or countries must undergo quarantine lasting at least 30 days with respect to major epizootic and notifiable diseases, such as bluetongue, bovine tuberculosis, brucellosis, and foot and mouth disease [6]. Paratuberculosis does not belong to the above mentioned group and the examination of imported ruminants therefore depends on the preference of the owner of the receiving farm. In addition, due to economic constraints, the belief that paratuberculosis does not represent a problem, or because of its low priority compared to other animal diseases, paratuberculosis control programmes in Europe are mostly based on voluntary participation [7]. In some countries, paratuberculosis numbers among the notifiable diseases, but there is no obligation to test animals with clinical signs. Thus, animals are not examined due to concerns regarding their obligatory elimination or problems with selling animal remains (herds with paratuberculosis status) [7].

Animals can be negatively influenced either by physical (hunger, thirst, injury, etc.) or psychological/ethological stress (handling, restraint, new environment, etc.). Compared to domesticated, calm animals, wild species with excitable temperaments react to stressors more sensitively [8]. While physical stress is connected with pain, psychological stress is characterised by fear which is one of the most important stressors [8]. Various stressors can detrimentally affect the immune system of animals [9], causing disruption of commensal microflora [10, 11], enhancing susceptibility to diseases [12], or activating latent infections [5].

In the best case scenario, animals are examined for the presence of MAP before their import into a new farm. Healthy animals diagnosed as MAP-negative are then released into the area of a new farm with other animals. The goal of this work was to assess the influence of stress connected with transport to a new farm in animals that were negative for MAP but which had originated from a herd in which MAP was previously detected. Although PCR is not able to distinguish between live and dead organisms, a key main advantage lies in its higher sensitivity compared to cultivation [13]. In the case of mouflons (sheep) in which cultivation is complicated and the presence of nongrowing isolates was described [14], real time quantitative PCR (qPCR) represents a sensitive tool for detection of MAP, based on copy number of IS*900* in MAP chromosome.

## 2. Material and Methods

### 2.1. The Mouflons and the Quarantine

The imported group of mouflons consisted of six individuals of different ages; two mouflon ewes with their offspring were included (Table 1). All animals came from a mouflon farm free of clinical paratuberculosis, although MAP DNA was found in the faeces of a few individuals (18.2% positivity; low concentrations between 10^1^ and 10^2^ MAP cells; data not shown) based on copy number of IS*900* in MAP chromosome. In the six animals selected, MAP DNA was repeatedly (twice) not detected in their faeces (IS*900* qPCR) four and two weeks before the transportation. All animals were transported from the farm of origin to the receiving farm in boxes used for animal transport (one hour journey). The experiment was conducted on an eleven hectare farm which consisted of about 70 heads of mouflons and 25 heads of fallow deer. The farm has for many years been subjected to monitoring for the presence of paratuberculosis. In the mouflons, the presence of MAP was previously demonstrated using culture and qPCR. However, at the time of the experiment, neither culture nor qPCR revealed MAP in the mouflons inhabiting the receiving farm.

After arrival, the mouflons were placed into a quarantine enclosure (circle ground plan, 50 m^2^) which consisted of an “open space” connected with a sheltered sleeve used for capturing animals and the collection of samples (“tunnel” connected with the enclosure and with reclosable partitions on both ends). Animals had unlimited access to the sleeve which provided them protection against unfavourable weather. The ground in the quarantine was covered with sand (not originating from the farm; 10 cm layer) and the mouflons were fed with MAP-free hay. The experiment lasted 17 days which included 12 days in the quarantine enclosure and subsequent five days when the mouflons were released into the area of the farm with other mouflons.

### 2.2. Sample Collection and DNA Isolation

During the quarantine enclosure period, faeces were collected using disposable gloves on each day. Seven times during quarantine, samples of blood were collected from each animal into a vial with EDTA (Amresco, Solon, OH, USA), and samples of milk were collected (on the same days as blood samples) from mother ewes (animals number 2 and 3; Table 1). All samples were transported to the laboratory at 4°C. DNA isolation from faeces was performed using a modified QIAamp DNA Stool Kit (Qiagen, Hilden, Germany) protocol [15]. According to the procedure described by Slana et al. [16], 15 mL of milk samples was used for DNA isolation. Blood was processed according to the instructions of a commercial Blood and Tissue Kit (Qiagen). The absence of MAP in hay used for feeding was confirmed using IS*900 *qPCR on DNA extracted using the PowerFood Microbial DNA Isolation Kit (MoBio, Carlsbad, CA, USA).

### 2.3. Detection of MAP Using qPCR

Detection of MAP DNA was based on the MAP-specific IS*900* sequence [17]. Each qPCR run included a calibration curve to allow calculation of the absolute number of MAP cells per gram or mL of the sample. The protocol included an internal amplification control to identify inhibitors in analysed DNA samples. Each sample was analysed in duplicate. All IS*900* qPCR analyses were performed on a LightCycler 480 Instrument (Roche Diagnostics).

### 2.4. Statistical Analysis

Statistical analysis was performed using the GraphPad Prism 5.04 (GraphPad Inc.) statistical software. The frequencies of occurrence of positive results in the quarantine and postquarantine periods were compared using Fisher's Exact Test.

## 3. Results

None of the animals showed overt clinical signs of disease or the presence of MAP in faeces before the transport. In spite of this, MAP DNA was found in the faeces of all animals either on the first or second day after their transport. During quarantine, MAP DNA was continuously found in the faeces, although the shedding for individual animals was irregular. The highest number of positive results (in days) was found for lamb number 5 and the second highest rate in its mother (number 3). After the release of animals, MAP was detected for a further two days, after which it was not detected for the subsequent three days, except for one positive case (lamb number 5; Table 1). Statistically significant differences were found between frequencies of MAP-positive animals (young and adult) in quarantine and after it and between adult animals in the quarantine and postquarantine periods (Table 2; *P* > 0.05). Odds ratios (odds of IS*900*-positive result in animals in quarantine compared to animals out of quarantine) were 3.3 and 3.1 for the group of adults and all animals (young and adults together), respectively.

Examination of blood was negative in all cases (Table 1). On two occasions, a very low MAP positivity was found in the milk of both ewes (Table 3). The hay used for feeding was demonstrated to be MAP-negative (data not shown).

## 4. Discussion

Various stress factors can significantly influence the microbial diversity of gut microflora [10, 11]. In addition, disturbing the balance and diversity of gut microflora can affect levels of pathogens in the gastrointestinal tract and lead to their shedding from subclinically infected animals [18]. In our case, handling, transport, a novel environment, and dietary changes could have induced the shedding of MAP in the mouflons (Tables 1 and 2), which were previously demonstrated to be clinically healthy and negative by PCR examination. These mouflons, which originated from a herd in which MAP was previously detected in a few animals using qPCR, must have been therefore challenged by MAP infection before the transport. These animals then harboured the latent form of paratuberculosis, which is characterised by an absence of MAP shedding in faeces and clinical symptoms. A similar effect was observed in clinically healthy cattle and wild deer in which stress probably activated latent malignant catarrhal fever [19, 20]. Freeman et al. [21] described the reactivation of *Herpes simplex* virus infection in mice after their exposure to restraint stress.

Generally, paratuberculosis characterised by a long incubation period is mostly induced by stress factors occurring during the life of animals, such as late pregnancy or early lactation [22]. Young animals are the most susceptible to MAP infection [5]. Lamb number 5 was the highest MAP shedder in this study. In lamb number 6, the frequency of MAP shedding was comparable with other animals (Table 1). However, due to the low age of the lambs (both 1/4 of year) and slow character of the disease, the detection of MAP in these lambs was probably caused by “passive pass-through” rather than by real infection. Thus, the demonstration of MAP in the lambs would most likely reflect the presence of MAP in other animals, particularly in their mothers.

The shedding in faeces was not regular in individual animals but was intermittent, which is characteristic for paratuberculosis. MAP shedding in milk is also known to be irregular and its likelihood is higher in symptomatic compared to asymptomatic animals [23, 24]. The proportion of MAP shed in faeces is generally higher compared to milk [25], which could explain why MAP was observed only twice in the milk of the two ewes over the examined period (Table 3). Blood samples were always negative which could be explained by the short-term influence of stress. This stress manifested as MAP shedding through faeces but not as systemic MAP bacteraemia, which occurs especially in animals in late stages of infection with accompanying clinical symptoms. Previously, MAP was detected in the blood of one bull which shed a high concentration of MAP in its faeces and showed clinical signs of the disease [26]. MAP was also detected in the blood of subclinical cows; however, the number of positive cases was significantly lower compared to animals in the clinical phase [27].

After the release of the animals from quarantine, MAP was still detected in faeces for two days, but then the excretion was undetectable. Except for one case (lamb number 5), no positive finding was made on the subsequent three days (Tables 1 and 2). Releasing the animals from quarantine together with their grazing on pasture instead of hay feeding could be probably the most important factors which decreased MAP shedding in the imported animals. Therefore, novelty, transport, novel environment, or change of feeding, together with the enclosure of animals in a confined and cramped space could be considered as the factors which induced the MAP shedding in this study. In this regard, the placement of imported animals into quarantine can help to distinguish truly healthy animals from those with latent MAP infection. The capture of animals for sample collection did not seem to impose a significant stress as almost all samples collected from animals released out of quarantine were negative (Tables 1 and 3).

## 5. Conclusions

In conclusion, as paratuberculosis does not belong to the group of major epizootic and notifiable diseases according to European legislation, animals transported to a new farm do not need to be examined for the presence of MAP or to undergo quarantine. However, in this study we observed MAP shedding from healthy mouflons with no clinical symptoms and previously determined as MAP-negative after their transport to a new farm. The results of this study call into question a sole reliance on examination of animals in their environment before transport and also underline the risk of admitting such animals onto new farms without previous placement in quarantine and further examination. Obviously, therefore, clinically healthy and diagnostically negative animals coming from exporting farms can potentially represent a risk of paratuberculosis for other animals in a receiving farm and its environment. For this reason, we recommend that animals be examined not only before the transport to a new farm but also after the transport. We suggest keeping animals in a quarantine enclosure until the results of the examination are known. Due to the intermittent shedding of MAP, at least two examinations are needed.

## Figures and Tables

**Table 1:** Number of *Mycobacterium avium* subsp. *paratuberculosis *cells (per gram) of the faeces of healthy mouflons imported to a new farm and determined by IS*900* real time quantitative PCR.

Day	Placement of animals	Animal number					
		1	2^a^	3^b^	4	5^b^	6^a^
		7-8 years old	4 years old	4 years old	1 year old	1/4 years old	1/4 years old
1	Quarantine	0	0	1.72 × 10^2^	4.79 × 10^2^	1.79 × 10^0^	0
2		9.58 × 10^1^	1.01 × 10^2^	1.96 × 10^2^	1.38 × 10^2^	2.65 × 10^2^	1.99 × 10^2^
3		2.20 × 10^2^	0	0	1.09 × 10^1^	3.50 × 10^1^	0
4		2.98 × 10^2^	0	0	0	1.06 × 10^2^	0
5		0	0	0	1.16 × 10^2^	1.68 × 10^2^	3.32 × 10^2^
6		0	7.62 × 10^1^	0	0	1.56 × 10^2^	0
7		6.18 × 10^2^	0	4.74 × 10^2^	0	0	3.74 × 10^1^
8		2.24 × 10^3^	7.93 × 10^2^	4.89 × 10^2^	0	1.03 × 10^2^	5.67 × 10^2^
9		9.69 × 10^2^	9.69 × 10^2^	9.69 × 10^2^	9.69 × 10^2^	9.69 × 10^2^	9.69 × 10^2^
10		1.53 × 10^2^	4.31 × 10^1^	7.96 × 10^2^	7.09 × 10^1^	8.33 × 10^2^	1.71 × 10^2^
11		0	0	3.59 × 10^1^	0	4.81 × 10^2^	0
12		0	0	0	0	0	0
13	Released out of quarantine	1.30 × 10^1^	0	2.85 × 10^0^	0	3.25 × 10^2^	8.57 × 10^1^
14		3.99 × 10^2^	2.86 × 10^1^	0	2.51 × 10^0^	7.60 × 10^2^	0
15		0	0	0	0	0	0
16		0	0	0	0	0	0
17		0	0	0	0	5.33 × 10^2^	0

^a^Mother and her offspring.

^b^Mother and her offspring.

**Table 2:** Percentages of *Mycobacterium avium* subsp. *paratuberculosis*-positive mouflons determined by real time IS*900* quantitative PCR.

Placement	Adult^a^ (%)	Young^b^ (%)	Adult + Young (%)
Quarantine	52.1	66.7	56.9
Out of quarantine	25.0	40.0	30.0

^a^Animals older than one year (animals numbers 1–4).

^b^Animals younger than one year (animals numbers 5 and 6).

**Table 3:** Number of *Mycobacterium avium* subsp. *paratuberculosis* cells (per millilitre) in the milk of healthy mouflon ewes transported to a new farm.

Animal number	Age	In quarantine				Released out of the quarantine		
		Day 1	Day 5	Day 7	Day 9	Day 13	Day 15	Day 17
2	4 years	3.28 × 10^0^	0	0	2.11 × 10^0^	1.00 × 10^0^	0	0
3	4 years	0	0	0	1.00 × 10^0^	3.07 × 10^0^	0	0
